# CT-based radiomic nomogram for preoperative prediction of DNA mismatch repair deficiency in gastric cancer

**DOI:** 10.3389/fonc.2022.883109

**Published:** 2022-09-16

**Authors:** Qingwen Zeng, Yanyan Zhu, Leyan Li, Zongfeng Feng, Xufeng Shu, Ahao Wu, Lianghua Luo, Yi Cao, Yi Tu, Jianbo Xiong, Fuqing Zhou, Zhengrong Li

**Affiliations:** ^1^ Department of Gastrointestinal Surgery, The First Affiliated Hospital, Nanchang University, Nanchang, China; ^2^ Institute of Digestive Surgery, The First Affiliated Hospital of Nanchang University, Nanchang, China; ^3^ Department of Radiology, The First Affiliated Hospital, Nanchang University, Nanchang, China; ^4^ Jiangxi Medical College, Nanchang University, Nanchang, China; ^5^ Department of Pathology, The First Affiliated Hospital of Nanchang University, Nanchang, China

**Keywords:** gastric cancer (GC), radiomics, microsatellite instability, nomogram, LASSO, DNA mismatch repair deficiency

## Abstract

**Background:**

DNA mismatch repair (MMR) deficiency has attracted considerable attention as a predictor of the immunotherapy efficacy of solid tumors, including gastric cancer. We aimed to develop and validate a computed tomography (CT)-based radiomic nomogram for the preoperative prediction of MMR deficiency in gastric cancer (GC).

**Methods:**

In this retrospective analysis, 225 and 91 GC patients from two distinct hospital cohorts were included. Cohort 1 was randomly divided into a training cohort (n = 176) and an internal validation cohort (n = 76), whereas cohort 2 was considered an external validation cohort. Based on repeatable radiomic features, a radiomic signature was constructed using the least absolute shrinkage and selection operator (LASSO) regression analysis. We employed multivariable logistic regression analysis to build a radiomics-based model based on radiomic features and preoperative clinical characteristics. Furthermore, this prediction model was presented as a radiomic nomogram, which was evaluated in the training, internal validation, and external validation cohorts.

**Results:**

The radiomic signature composed of 15 robust features showed a significant association with MMR protein status in the training, internal validation, and external validation cohorts (both *P*-values <0.001). A radiomic nomogram incorporating a radiomic signature and two clinical characteristics (age and CT-reported N stage) represented good discrimination in the training cohort with an AUC of 0.902 (95% CI: 0.853–0.951), in the internal validation cohort with an AUC of 0.972 (95% CI: 0.945–1.000) and in the external validation cohort with an AUC of 0.891 (95% CI: 0.825–0.958).

**Conclusion:**

The CT-based radiomic nomogram showed good performance for preoperative prediction of MMR protein status in GC. Furthermore, this model was a noninvasive tool to predict MMR protein status and guide neoadjuvant therapy.

## Introduction

Gastric cancer (GC) is one of the most common malignant diseases and ranks as the fourth leading cause of cancer-related death worldwide (1). According to 2020 statistics, the incidence and mortality of GC both ranked third among solid tumors in China (1). The first diagnosis of GC patients with locally advanced disease is approximately two-thirds, so most guidelines recommend comprehensive therapy as the standard treatment method, mainly including neoadjuvant therapy plus surgery (2, 3). Kim et al. found that GC patients with cStage III disease with microsatellite instability-high (MSI-H) had better survival than those with microsatellite stability (MSS) after neoadjuvant chemotherapy (4). A meta-analysis of four randomized clinical trials of adjuvant chemotherapy based on immunotherapy in GC showed that the overall survival of GC patients with microsatellite instability (MSI) was significantly better than that of patients with MSS (hazard ratio [HR], 0.69; 95% CI, 0.55 to 0.88; P = 0.003) (5). However, An et al. showed that in MSI-H patients with stage II or III GC, adjuvant chemotherapy based on 5-FU did not receive any benefit, which gives a guideline that these patients are not suitable for the 5-FU-based chemotherapy drugs (6). MSI is caused by a lack of DNA mismatch repair protein deficiency (dMMR), which accounts for 6%–25% of GC patients (7). Interestingly, MSI creates a high mutation burden, increases the number of neoantigens in tumor tissues, and these individuals exhibit high levels of immune checkpoint molecules (8, 9). As a result, comprehensive therapy based on anti-PD-1/-L1 Abs may be a good option for MSI GC patients. Thus, assessing the MMR status of all GC patients is a level I recommendation in the current guidelines.

In clinical practice, immunohistochemistry (IHC) or DNA detection is the primary technology to evaluate MSI using postoperative tumor tissues. Although preoperative gastroscopy tumor samples could also be used to detect MSI, sampling bias and poor DNA quality may lead to misleading findings (10, 11). Thus, there is insufficient evidence to choose an appropriate neoadjuvant therapy for patients who are suffering from locally advanced GC. Furthermore, gastroscopic biopsy is a procedure that requires good physical condition for the patient, but it cannot be conducted on patients who have inadequate circumstances, including poor coagulation, cardiopulmonary dysfunction, and unacceptable gastroscopy. In some primary hospitals, there is difficulty in implementing these technologies. Therefore, developing a relatively non-invasion and acceptable strategy for detecting the MMR status of GC patients is an urgent task (12).

In comparison to gastroscopic biopsy and surgery without invasive injury, computed tomography (CT) is a noninvasive technology commonly used for the diagnosis, response evaluation, and postoperative follow-up of gastric cancer (13). Prior studies have demonstrated that quantitative radiomic features of CT images are associated with elements of the tumor microenvironment, such as the tumor stroma, gene expression level, and even tumor-infiltrating lymphocytes (11, 14, 15). Referring to the immunohistochemistry scores for α-smooth muscle actin and periostin, Yuming et al. built a deep-learning model to precisely assess tumor stroma using CT images in GC, which can guide treatment decisions and predict prognosis for patients (11). Human epidermal growth factor receptor 2 (HER2) status may be accurately predicted using a radiomic nomogram that combines a radiomic signature and carcinoembryonic antigen level (CEA) (16). Currently, Okihide et al. utilized five clinicopathological features (age, sex, location, T stage, and distant metastasis) to predict MSI (AUC = 0.82, 95% CI: 0.75–0.87) in GC. However, the main collecting clinicopathological features are derived from postoperative gastrectomy (17). As can be observed, predicting MMR status of GC patients based on clinicopathological features falls well short of clinical diagnostic standards. Furthermore, MSI was associated with tumor location, size and lymph node status in GC CT images (18). According to the above results, constructing a prediction model based on radiomic features may produce the desired result for the MSI diagnosis.

Although model-based radiomic features perform well in the identification of MMR deficiency in colorectal cancer (8, 19), this is the first study to employ radiomic features to predict MMR status in GC. This study aimed to develop and validate a CT-based radiomic nomogram for the preoperative prediction of MMR deficiency in GC.

## Materials and methods

### Patients

**Figure 1:**
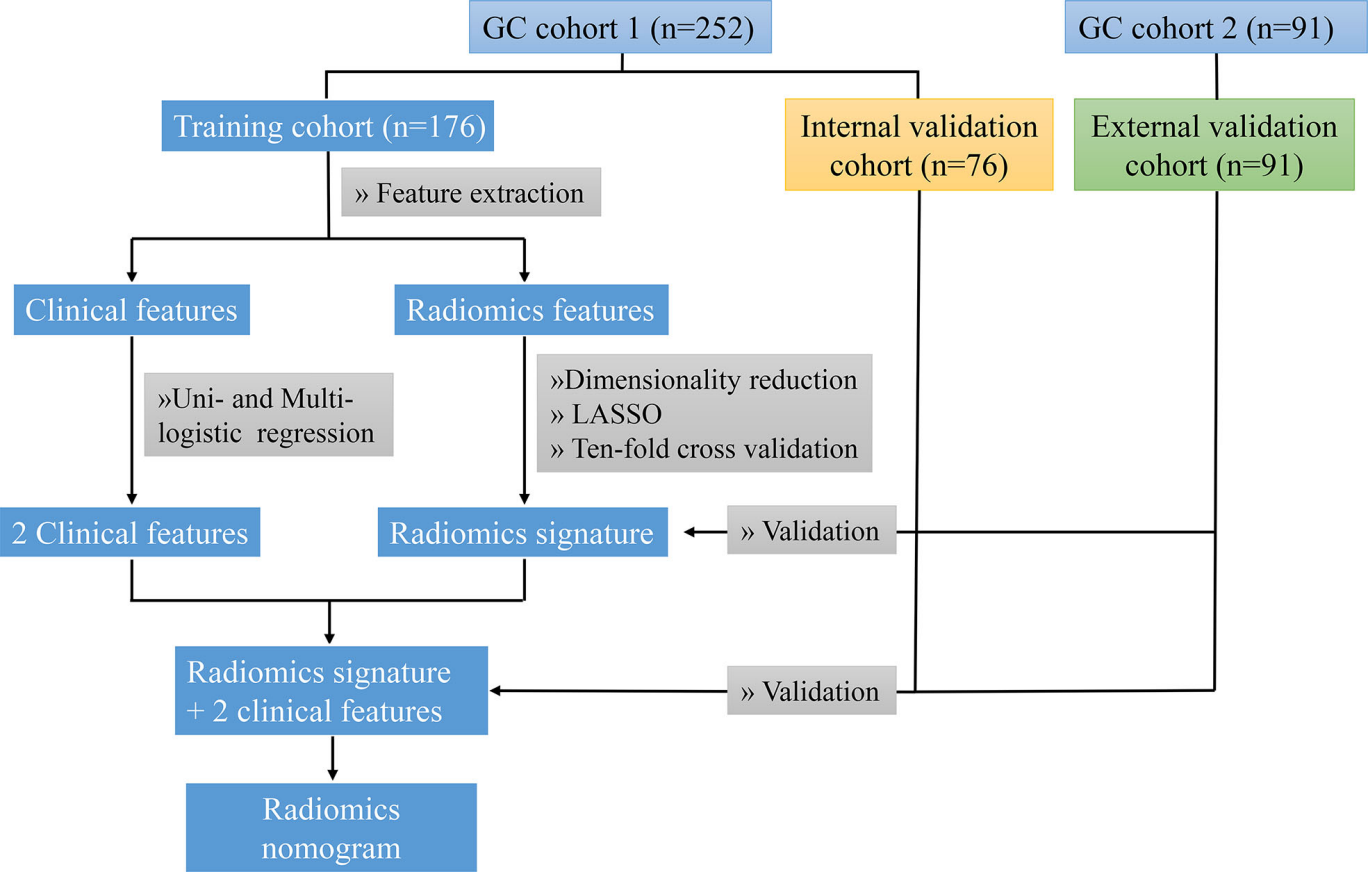
The technology roadmap represents workflow in this study. The GC cohort 1 was collected from the First Affiliated Hospital of Nanchang University (Donghu hospital), and the cohort 2 was collected from the First Affiliated Hospital of Nanchang University (Xianghu hospital).

This retrospective study was approved by the Ethics Committee of the First Affiliated Hospital of Nanchang University and the patients. Cohort 1 included 252 GC patients who underwent radical gastrectomy were enrolled in this study with preoperative contrast-enhanced CT examination from June 2018 to December 2021 at the First Affiliated Hospital of Nanchang University (Donghu Hospital). Another cohort 2 collected 91 GC patients from April 2020 to December 2021 at the First Affiliated Hospital of Nanchang University (Xianghu Hospital). The inclusion criteria were as follows (1): histologically proven diagnosis of GC; (2) preoperative contrast-enhanced CT within a month; (3) MMR protein status tested by IHC; and (4) no preoperative adjuvant therapy. The exclusion criteria were as follows: (1) any preoperative adjuvant therapy; (2) poor quality CT images: poor filling of the stomach with unsatisfactory gastric distention and substantial motion artifacts; and (3) lack of clinical data. Cohort 1 was randomly divided into the training (n = 176) and internal validation (n = 76) cohorts at a rate of 7:3 (**Figure 1**). The training cohort contained proficient DNA mismatch repair (pMMR, n = 105) and dMMR (n = 71). The internal validation cohort contained pMMRs (n = 46) and dMMRs (n = 30). Cohort 2 was used as an external validation cohort, which contained pMMR (n = 64) and dMMR (n = 27).

The collected preoperative clinical characteristics of the patients included age, body mass index (BMI), sex, tumor location, CEA status (normal or abnormal), CA19-9 status (normal or abnormal), CA12-5 status (normal or abnormal), AFP status (normal or abnormal), and CT-reported T stage (T1, T2, T3, T4) and N stage (N0, Nx). The normal range of CEA, CA19-9, CA12-5, and AFP was, respectively, 0–6.5 ng/ml, 0–27 U/ml, 0–35 U/ml, and 0–7 ng/ml. Additionally, we measured several semantic features to compare the difference in predictive level with radiomic features, including long diameters of the tumor, short diameters of the tumor, tumor thickness, and CT value of the tumor in the portal phase. The reference for CT imaging classification of gastric cancer was based on the Chinese Society of Clinical Oncology (CSCO): Clinical guidelines for the diagnosis and treatment of gastric cancer, 2021 (20). Reference for CT imaging classification of gastric cancer can be referred to **Supplementary Figure 1**.

### MMR protein status evaluation

To determine MMR protein status, we employed IHC to test four correlated proteins, including mutL homolog 1 (MLH1), mutS homolog 2 (MSH2), mutS homolog 6 (MSH6) and mismatch repair system component (PMS2). According to MSI status, GC patients were divided into three groups: MSI-H, MSI-L, and MSI stability (MSS). The expression level of MMR proteins was used to diagnose MSI status. The positive staining of all four proteins represented MSS/MSI-L (pMMR), but the MMR proteins with anyone assessed as negative represented MSI-H (dMMR) (21).

### CT image acquisition

Before abdominal contrast-enhanced CT, all patients received Racanisodamine Hydrochloride injection 20 mg *via* intramuscular injection and drank 1,000–2,000 ml of water. The picture archiving and communication system (Carestream, Canada) was used to export CT images of the portal venous phase. Contrast-enhanced CT scanning of cohort 1 was performed using a 192-channel CT (Siemens Healthcare) in Donghu hospital. Cohort 2 was scanned by 256-channel CT (Siemens Healthcare) and 320-channel CT (Aquilion ONE) in Xianghu hospital. The acquisition parameters were as follows: tube voltage of 80 to 120 kVp; tube current of 120–300 mAs; the pitch of 0.6 to 1.25 mm; an image matrix of 512 × 512; and reconstruction slice thickness of 1 or 2 mm. After intravenous injection of contrast media (1.5 ml/kg, at a rate of 2.5–3.5 ml/s), the arterial phase and portal venous phase were acquired within 25–30 s and 65–70 s, respectively.

### Radiomic features extraction

**Figure 2:**
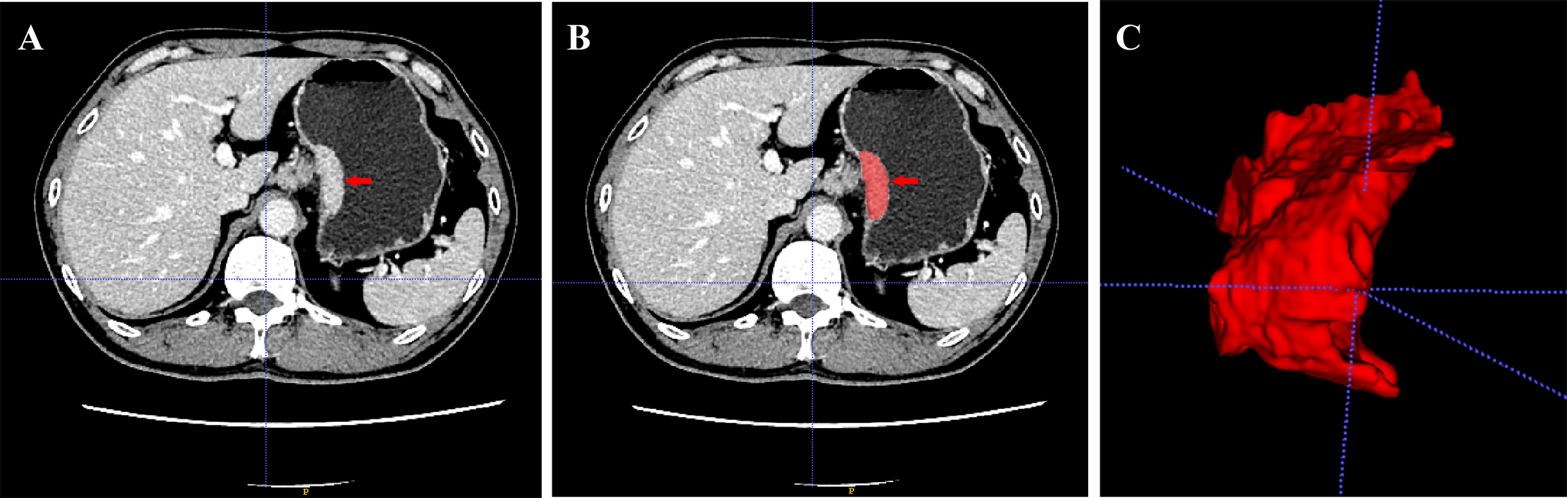
Manual segmentation of tumor with a GC patient. **(A)** A slice portal venous phase of contrast-enhanced CT images of the tumor. **(B)** the red label masks a slice CT image of the tumor with manual segmentation. **(C)** Three-dimensional (3D) image of the tumor with manual segmentation.

The extent of the tumor lesion was enhanced and more easily distinguished between the tumor and peripheral normal tissue during the portal venous phase, and many previous studies used this phase to segment tumor lesions (22, 23). In this study, we employed ITK-SANP software (version 3.6.0, USA) to manually segment regions of interest (ROIs) (**Figure 2**). Lesions were located by significantly enhanced parts and thickening of the gastric wall for incorporation with the clinical characteristics of pathology specimens (24). The ROIs were manually drawn carefully to highlight neighboring upper and lower slices of the solid tumor, while we were careful to avoid involving the normal gastric wall and nearby air or fluid (8). Radiologist 1 (Zhu with 5 years of experience) delineated the ROI of all 343 GC patients. We randomly selected 30 patients, re-drew their ROIs for feature extraction by Zhu one month later, and analyzed the result to prevent intraobserver differences from affecting the reproducibility of radiomic features. To confirm the interobserver reproducibility, a second radiologist (Zhou, who has 10 years of experience) delineated the ROIs for these 30 patients (16). Radiomic features were extracted using PyRadiomics software (version 2.2.0) (25). Finally, eight hundred and fifty-one radiomic features were extracted and classified into four categories: shape, size, texture, and wavelet (**Supplementary Table S1**).

### Radiomic feature selection and radiomic signature establishment

Intra- and interclass correlation coefficients (ICCs) were used to evaluate the reproducibility and robustness of the extracted radiomic features. Only radiomic features with an ICC ≥0.8 were considered highly stable and retained for subsequent analysis. A Z-score normalization was used to standardize the radiomic feature data in the training, internal validation, and external validation cohorts. Then, we employed the Mann–Whitney U test to identify significantly different features between the pMMR and dMMR groups, with P <0.05 in the training cohort (15). The least absolute shrinkage and selection operator (LASSO) regression was used for feature selection and radiomic signature construction in the training cohort. We used 10 cross-validations to define the regularization parameter λ. Finally, the radiomic score (Rad-score) was developed and demonstrated as a formula that was calculated by determining a linear combination of the selected features and the product of their respective coefficients. The R software package “Glmnet” was used for LASSO logistic regression.

The discriminative ability of the radiomic signature for predicting MMR deficiency was based on the receiver operating characteristic (ROC) curve and the area under the curve (AUC).

### Establishment of the radiomic nomogram

A univariate logistic regression analysis was used to investigate the correlation between MMR deficiency and clinical characteristics in cohort 1 GC patients. Multivariate logistic regression analysis was used to build a prediction model by incorporating radio-score and clinical characteristics with *P*-values <0.05 in the univariate logistic regression analysis. The chosen features with *P*-values <0.05 in the multivariable analysis were used to build a radiomics-based model, which was presented as a radiomic nomogram in the training cohort. The ROC curve was applied to evaluate the discriminative performance of the radiomic nomogram in training, internal validation, and external validation cohorts. A calibration curve was applied to evaluate the radiomic nomogram in all cohorts. To estimate the clinical usefulness of the prediction models, decision curve analysis (DCA) was performed to assess the net benefit of the radiomic nomogram and signature in the training, internal validation, and external validation cohorts.

### Statistical analysis

In this study, we employed IBM SPSS Statistics (Version 20.0, USA) to assess the clinical data. The t-tests or the Mann–Whitney U-test were used to compare the numerical data (age and BMI), while the Chi-square or Fisher tests were used to compare the categorical data (sex, sex, tumor location, CEA, CA19-9, CA12-5, AFP level, CT-reported T stage, and N stage) in the training, internal validation, and external validation cohorts. Furthermore, the t-test or the Mann–Whitney U-test was used to assess the correlation between radiomic features and MMR status in the training cohort, which was the first dimensionality reduction. The R software (version 3.3.1, Austria; http://www.R-project.org) was used to study the radiomic feature data and build a prediction model. A *P*-value of <0.05 was defined as statistically significant.

## Results

### Patients’ clinical characteristics

The characteristics of GC patients are presented in **Table 1**. Cohort 1 was randomly divided into a training cohort (n = 176, average age: 62 years old; range: 23–87 years old) with 107 males and 69 females, and an internal validation cohort (n = 76, average age: 62 years old; range: 30–83 years old) with 50 males and 26 females. There were 91 GC patients, 55 males and 36 females (average age: 61 years old; range: 37–78 years old) in the external validation cohort. In the training cohort, statistically significant differences in age, sex, tumor location, CEA level, and CT-reported T stage were identified between pMMR and dMMR patients (*P*-value <0.05), while other clinical features (BMI, CA19-9 level, CA125 level, AFP level, and CT-reported N stage) showed no statistically significant differences (*P*-value >0.05). Furthermore, we found that age and CT-reported N stage showed statistically significant differences between pMMR and dMMR patients in the internal validation cohort. There were also only two clinical features (sex and CEA level) that had significant differences in the external validation cohort.

**Table 1:** Characteristics of GC patients in training, internal validation and external validation cohorts.

Characteristics	Training cohort (n = 176)		*P*-value	Internal validation cohort (n = 76)		*P*-value	External validation cohort (n = 91)		*P*-value
	pMMR	dMMR		pMMR	dMMR		pMMR	dMMR
**Age (year)**	59.70 ± 9.91	65.94 ± 11.47	**<0.001**	58.15 ± 11.72	64.23 ± 12.18	**0.033**	61.72 ± 8.15	61.30 ± 11.29	0.828
**BMI**	22.10 ± 3.39	21.98 ± 3.47	0.825	22.29 ± 3.08	22.40 ± 2.81	0.884	22.54 ± 2.93	22.80 ± 3.68	0.714
**Sex**			**0.024**			0.715			**0.001**
Male	71	36		31	19		46	9	
Female	34	35		15	11		18	18	
**Tumor location**			**0.039**			0.855			0.558
Upper-third	31	10		11	6		15	4	
Middle-third	26	17		10	8		18	10	
Lower-third	48	44		25	16		31	13	
**CEA level**			**0.003**			0.299			**0.030**
Normal	87	69		38	28		54	27	
Abnormal	18	2		8	2		10	0	
**CA19-9 level**			0.617			0.694			0.719
Normal	86	56		37	23		50	22	
Abnormal	19	15		9	7		14	5	
**CA12-5 level**			0.781			1.000			0.579
Normal	98	67		44	29		62	25	
Abnormal	7	4		2	1		2	2	
**AFP level**			0.722			0.153			0.508
Normal	101	69		46	28		63	26	
Abnormal	4	2		0	2		1	1	
**CT-reported T stage**			**<0.001**			0.258			0.141
T1	18	11		7	6		6	2	
T2	16	12		4	3		7	4	
T3	32	19		14	14		19	14	
T4	39	29		21	7		32	7	
**CT-reported N stage**			0.089			**0.019**			0.647
N0	42	45		18	20		48	19	
N1 + N2 + N3	63	26		28	10		16	8	
**Rad-scores**	−2.36 ± 2.63	1.01 ± 1.73	**<0.001**	−3.52 ± 4.00	1.67 ± 1.57	**<0.001**	−2.33 ± 3.80	0.93 ± 1.24	**<0.001**

pMMR, proficient DNA mismatch repair; dMMR, deficient DNA mismatch repair; BMI, body mass index; CEA normal range: 0–6.5 ng/ml; CA19-9 normal range: 0-27 U/ml; CA12-5 normal range: 0-35 U/ml, AFP normal range: 0-7 ng/ml. The bolded P-value showed statistically significant (P-value<0.05).

### Radiomic signature establishment

**Figure 3:**
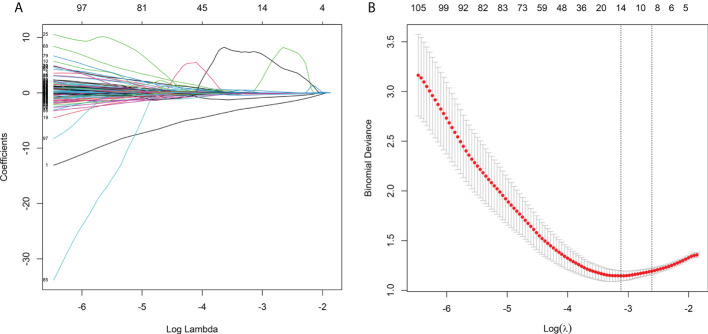
Feature selection using LASSO logistic regression and the least absolute shrinkage. **(A)** LASSO coefficient profiles of the features. Different color line shows the corresponding coefficient of each feature. **(B)** Tuning parameter (λ) selection in LASSO model. The first vertical line was drawn *via* ten-fold cross-validation based on minimum criteria.

Of eight hundred and fifty-one radiomic features extracted from the delineated ROIs, 49 features with ICCs <0.8 were excluded (**Supplementary Table S2**). A total of 802 radiomic features were found to be substantially different between pMMR and dMMR patients, and they were used to build a radiomic signature *via* least absolute shrinkage and selection operator regression with tenfold cross-validation. Finally, 15 radiomic features were chosen to evaluate the Rad-score of each GC patient (**Supplementary Table S3**). The difference in Rad-scores was statistically significant between pMMR and dMMR patients in training, internal validation, and external validation cohorts (*P*-value <0.001). The radiomic signature in the training cohort is depicted in **Figure 3**.

**Figure 4:**
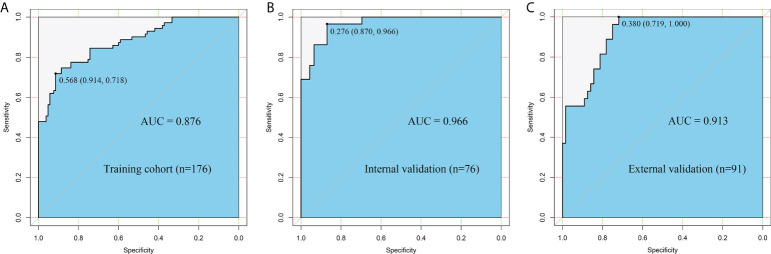
The ROC curves of the radiomic signature in the **(A)** training cohort, **(B)** internal validation cohort, and **(C)** external validation cohort.

The Rad-score of the dMMR group was significantly higher than that of the pMMR group in training, internal validation, and external validation cohorts. The AUC of the radiomic signature for the training cohort was 0.876 (95% CI: 0.824–0.928) (**Figure 4A** **)**. The training cohort had a sensitivity of 77.4%, a specificity of 83.8%, and an accuracy of 81.3%. In the internal validation cohort, the AUC of the radiomic signature was higher than that of the training cohort (AUC = 0.966, 95% CI: 0.933–0.999) (**Figure 4B** **)**. The internal validation cohort had a sensitivity of 75.9%, a specificity of 95.7%, and an accuracy of 88.0%. Furthermore, the AUC of the radiomic signature for the external validation cohort was 0.913 (95% CI: 0.857–0.969) with a sensitivity of 74.1%, specificity of 84.4%, and accuracy of 81.3% (**Figure 4C**).

### The performance difference between CT features and selected radiomic features to predict MMR status

To compare the performance between semantic features and 15 selected radiomic features to predict MMR status, we constructed predictive models, respectively. The highest AUC values of semantic features were 0.64 (95% CI: 0.52–0.75) and 0.64 (95% CI: 0.53–0.74) in the internal validation and external cohorts (**Table 2**). However, 15 selected radiomic features showed significantly better performance, with the highest AUC values of 0.82 (95% CI: 0.72–0.90) and 0.71 (95% CI: 0.60–0.80) in the internal validation and external cohorts (**Table 3**). The AUC value of the radiomic signature was also significantly higher than the combined CT features model in the internal validation and external cohorts.

**Table 2:** The performance of CT features extracted by radiologist to predict MMR status.

Semantic features	AUC (95%CI)	
	Internal validation cohort	External validation cohort
Long diameters of tumor (mm)	0.57 (0.45–0.69)	0.63 (0.52–0.73)
Short diameters of tumor (mm)	0.54 (0.42–0.65)	0.55 (0.44–0.66)
Tumor thickness (mm)	0.53 (0.42–0.65)	0.58 (0.47–0.68)
CT value of tumor in PP (HU)	0.53 (0.41–0.65)	0.64 (0.53–0.74)
Location (up and mid vs low)	0.53 (0.42–0.65)	0.57 (0.46–0.67)
CT-reported N stage (N0 vs Nx)	0.64 (0.52–0.75)	0.52 (0.42–0.63)
CT-reported T stage	0.60 (0.49–0.71)	0.60 (0.49–0.70)
Combined semantic features model	0.63 (0.49–0.76)	0.53 (0.39–0.66)

MMR status. 95% CI, 95% confidence interval.

**Table 3:** The performance of selected radiomic features to predict MMR status.

Radiomics features	AUC (95% CI)	
	Internal validation cohort	External validation cohort
Original shape elongation	0.82 (0.72–0.90)	0.68 (0.57–0.77)
Original shape flatness	0.61 (0.49–0.72)	0.57 (0.45–0.66)
Original shape surface area	0.68 (0.57–0.79)	0.59 (0.48–0.69)
Original glcm Imc2	0.72 (0.60–0.82)	0.68 (0.57–0.77)
Wavelet LHL glcm cluster shade	0.56 (0.44–0.67)	0.58 (0.48–0.69)
Wavelet LHL glcm cluster tendency	0.61 (0.50–0.72)	0.69 (0.59–0.78)
Wavelet LHL glcm Idn	0.67 (0.56–0.78)	0.54 (0.43–0.64)
Wavelet HLL glcm Idn	0.66 (0.55–0.77)	0.59 (0.48–0.69)
Wavelet LHL glrlm run entropy	0.65 (0.53–0.76)	0.70 (0.60–0.79)
Wavelet LHH first order 10 percentile	0.53 (0.41–0.64)	0.59 (0.48–0.69)
Wavelet HHH first order total energy	0.66 (0.54–0.76)	0.61 (0.50–0.71)
Wavelet HHL first order total energy	0.65 (0.53–0.76)	0.63 (0.52–0.73)
Wavelet HLH glszm small area high gray level emphasis	0.69 (0.58–0.79)	0.68 (0.57–0.77)
Wavelet LHL gldm small dependence emphasis	0.56 (0.44–0.67)	0.71 (0.60–0.80)
Wavelet HHL glrlm low gray level run emphasis	0.67 (0.55–0.77)	0.63 (0.52–0.73)
Radiomics signature	0.97 (0.93–1.00)	0.91 (0.86–0.97)

95% CI, 95% confidence interval.

### Construction of radiomic nomogram

**Figure 5:**
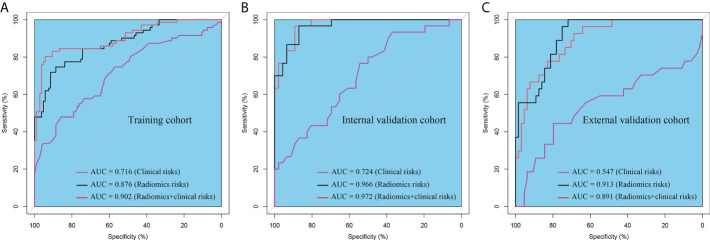
The ROC curves of the clinical risk, radiomic signature and radiomic nomogram (radiomic signature + clinical risk) in the **(A)** training cohort, **(B)** internal validation cohort, and **(C)** external validation cohort.

**Figure 6:**
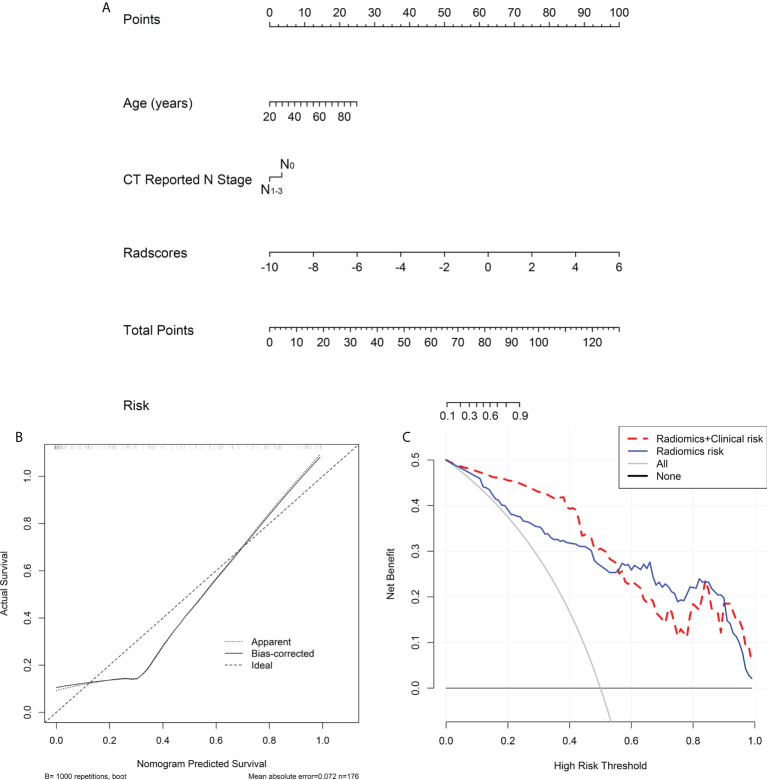
Radiomics nomogram developed with ROC, calibration curves, and decision curve analysis (DCA). **(A)** A radiomic nomogram was constructed in the training cohort *via* radiomic signature, age and CT reported N stage. **(B)** Calibration curve of the radiomic nomogram in the training cohort. **(C)** DCAs for radiomic nomogram and signature in the training cohort.

In the univariate and multivariate logistic regression analyses, age, CT-reported N stage, and Rad-score were independent predictors for assessing MMR status. In the univariate analysis, sex and CEA level were significantly correlated with MMR status, while no statistically significant correlation was found in the multivariate analysis (**Table 4**). Then, we used age, CT-reported N stage, and the Rad-score to build a radiomic nomogram to predict MMR status in the three cohorts (**Figure 6A**). The radiomic nomogram showed good performance for predicting MMR status in the training cohort with an AUC of 0.902 (95% CI: 0.853–0.951), in the internal validation cohort with an AUC of 0.972 (95% CI: 0.945–1.000), and in the external validation cohort with an AUC of 0.891 (95% CI: 0.825–0.958) (**Figure 5**). The training cohort showed a sensitivity of 80.3%, a specificity of 91.4%, and an accuracy of 86.9%. The internal validation cohort had a sensitivity of 70.0%, a specificity of 97.8%, and an accuracy of 86.8%. The external validation cohort had a sensitivity of 77.8%, a specificity of 81.3%, and an accuracy of 80.2%. The calibration curve of the radiomic signature and nomogram of three cohorts is presented in **Figure 6B** and **Supplementary Figure 2**, suggesting that the prediction model was acceptable. The DCA showed that the radiomic signature and nomogram would offer a more net benefit than either the default of all dMMR or non-dMMR in the three cohorts (**Figure 6C** and **Supplementary Figure 2**).

**Table 4:** Univariate and multivariate logistic regression analysis of risk factors of MMR status.

Variable	Univariate Logistic Regression		Multivariate Logistic Regression	
	OR (95% CI)	*P* value	OR (95% CI)	*P* value
Sex (male *vs* female)	0.57 (0.34–0.97)	**0.036**	0.49 (0.21–1.12)	0.094
Age	1.05 (1.02–1.08)	**<0.001**	1.05 (1.01–1.09)	**0.014**
BMI	0.99 (0.92–1.07)	0.900		
CEA level (normal vs abnormal)	0.20 (0.07–0.58)	**0.003**	2.94 (0.82–10.50)	0.097
CA19-9 level (normal vs abnormal)	1.22 (0.65–2.28)	0.528		
CA12-5 level (normal vs abnormal)	0.82 (0.26–2.52)	0.732		
AFP level (normal vs abnormal)	1.51 (0.37–6.20)	0.563		
Location (up and mid vs low)	1.31 (0.90–1.90)	0.146		
CT-reported N stage (N0 vs Nx)	0.36 (0.21–0.61)	**<0.001**	2.30 (1.04–5.07)	**0.038**
CT-reported T stage	0.94 (0.75–1.19)	0.654		
Rad-scores	3.23 (2.38–4.38)	**<0.001**	2.98 (2.18–4.08)	**<0.001**

OR, odds ratio; 95% CI, 95% confidence interval; BMI, body mass index; CEA normal range: 0–6.5 ng/ml; CA19-9 normal range: 0–27 U/ml; CA12-5 normal range: 0–35 U/ml, AFP normal range: 0–7 ng/ml. The bolded P-value showed statistically significant (P-value <0.05).

## Discussion

In this study, we developed and validated a prediction model to assess the MMR status of GC patients based on a radiomic signature and two clinical features: age and CT-reported N stage. The radiomic nomogram performed well in predicting MMR status in the training (AUC = 0.902), internal validation (AUC = 0.972), and external validation (AUC = 0.891) cohorts.

An increasing number of studies have confirmed that MSI-H or MMR deficiency is a remarkable biomarker for the diagnosis, treatment, and prognosis of GC patients (26, 27). MSI is defined as a phenotype of high mutation genomic MS, which is on account of MMR deficiency. Currently, MSI or MMR deficiency is detected by IHC and PCR-based molecular testing using tumor tissue after gastrectomy (28, 29). However, postoperative pathological results did not give timely advice on neoadjuvant therapy for individuals with locally advanced GC. Although preoperative gastroscopy can sample tumor tissue for testing MMR status, two limitations remain: histological assessment is also impacted by tumor tissue dynamic progression and geographic heterogeneity. Ottini et al. confirmed the heterogeneity of intratumoral MSI patterns observed in GC biology by assessing the microsatellite allele pattern in various sections of the same tumor studied (30). Similarly, Mathiak et al. showed that a biphasic MSH2 expression status in the same GC neoplasm (5%–23% of the tumor area was MSS and 85% MSI) (31). Radiomics features extracted from CT images were used in this study to assess the whole tumor and were easily repeated throughout the treatment period with no invasion. Previous studies showed that dMMR GC was significantly associated with CT semantic features, including a lower location, fewer lymph nodes, and smaller tumor thickness, implying that dMMR may be evaluated *via* radiomic features (18). To our knowledge, this is the first study to assess the potential of radiomic features to predict MMR status in GC based on preoperative clinical characteristics. Our study confirmed that the radiomic signature based on CT images performed well in predicting the MMR status of GC in the training (AUC = 0.876, 95% CI: 0.824–0.928), internal validation (AUC = 0.966, 95% CI: 0.933–0.999), and external validation cohorts (AUC = 0.913, 95% CI: 0.857–0.969). In comparison to colorectal cancer research, our prediction model appears to have greater diagnostic power for assessing MMR GC status (8, 19). Furthermore, a gastroscopic biopsy or surgery is a procedure that requires good physical condition for the patient, but it cannot be conducted on patients who have inadequate circumstances. Thus, this prediction model was a useful supplement strategy for predicting the MMR status of GC with a relatively non-invasion technique.

Radiomics converts medical pictures into mineable data by high-throughput extraction of numerous quantitative based on shape, size, volume, and other factors, which has proved useful in the investigation of diseased conditions (32, 33). Radiomic features differ from traditional semantic features of medical images extracted by radiologists in that they contain more messages about tumors and are more objective (33). In the radiomic signature, elongation represented the best independent risk to predict MMR status in GC with an AUC of 0.82 (95% CI: 0.72–0.90) in the internal validation cohort. Likewise, shape-related radiomic features, such as elongation, flatness, and surface area, outperformed semantic shape features extracted by radiologists. Several similar studies confirmed that elongation, flatness, standard deviation, skewness, kurtosis, and tumor contrast were promising radiomic features for gene expression prediction (34, 35). In particular, elongation and flatness features showed better identification of high Ki-67 expression in adrenocortical carcinoma by the Spearman rank method (36). The remaining independent predictors of radiomic features were 11 wavelet features and one gray level co-occurrence matrix feature. The AUC value of the radiomic signature was significantly higher than the combined CT features model in the internal validation and external cohorts with 0.97 (95% CI: 0.93–1.00) and 0.91 (95% CI: 0.86–0.97). Several previous researches demonstrated that wavelet features were significantly correlated with heterogeneity indices at the cellular level, which were promising radiomic features to evaluate prognosis in colorectal liver metastases patients (37). In this study, the radiomic signature that we constructed showed a reliable model to predict MMR status in GC, outperforming traditional semantic features extracted by radiologists.

Additionally, many studies have focused on the correlation between MMR status and different clinical features, which can be used to discriminate molecular expression levels and give individualized therapeutic guidance (38, 39). Previous studies showed that dMMR GCs were significantly correlated with female sex, advanced age, distal location, and intestinal type (40, 41). Martinez and coworkers discovered that GC patients with dMMR showed an earlier clinical stage (TNM stage I or II) and Borrmann type I or II, while they were initially diagnosed (21). In our studies, we found that the dMMR phenotype was also significantly associated with older age and fewer CT-reported lymphatic metastases. However, no association was detected between dMMR and CEA, CA19-9, CA12-5, or AFP levels in the blood tumor markers. Furthermore, Yexing and colleagues built a radiomic nomogram based on the radiomic signature and clinical features that performed well in determining HER2 status (16). We employed age, CT-reported N stage, and the Rad-score to develop a radiomic nomogram to predict MMR status. The radiomic nomogram showed good performance for predicting MMR status in the training cohort with an AUC of 0.902 (95% CI: 0.853–0.951), in the internal validation cohort with an AUC of 0.972 (95% CI: 0.945–1.000), and in the external validation cohort with an AUC of 0.891 (95% CI: 0.825–0.958). Okihide et al. constructed a clinical features model to predict dMMR, which showed lower evaluating capability with an AUC of 0.82 (95% CI: 0.75–0.87) and the model was not tested by the validation cohort, which may make the model unrepresentable (17). Therefore, our radiomic nomogram can efficiently discriminate dMMR GCs using a radiomic signature and clinical features in the preoperative.

Currently, radiomics-based GC research has focused on preoperative lymph node metastasis, Lauren categorization, the tumor immune milieu, genetic subtypes, and GC prognosis prediction (42–47). Identifying dMMR is crucial in our research since it guides preoperative clinical management for GC patients. Firstly, dMMR seems to be a biomarker for GC, which was associated with less lymphatic metastasis and an earlier T stage (41). Secondly, GC patients with confirmed MMR status are extremely important in clinical practice for guiding adjuvant and perioperative treatment (48, 49). A 1,990 GC patient study showed that dMMR GCs did not have better benefits in terms of disease-free survival (DFS) than pMMR GCs following R0 resection (6). When GC patients were treated only with surgery vs groups treated with chemotherapy, stage II or III GCs with dMMR status were correlated with better overall survival (OS) (50). The above results were confirmed by a multinational meta-analysis, which showed that GC patients with pMMR benefit from surgery plus chemotherapy rather than dMMR (51). Thirdly, MMR status might be associated with a response to immune checkpoint inhibitors in GC patients. A meta-analysis including 2,545 GC patients (including phase III KEYNOTE-062, CheckMate-649, JAVELIN Gastric 100, and KEYNOTE-061) revealed that GC patients with dMMR should be identified as a highly immunosensitive and specific subgroup for anti-PD-1 therapy (5), because of their intrinsic mutational burden-activated expression of immune checkpoints and inflammation (4, 52). Therefore, when patients are diagnosed, their MMR status must be accurately identified in order to provide a customized therapeutic schedule.

In this study, the main limitation is the retrospective nature of the study, which might have resulted in selection bias. However, we first built and validated a radiomic nomogram to assess the MMR protein status of GC patients based on the radiomic signature and clinical features. Secondly, because the distinction between tumor tissue and adjacent normal gastric tissue can be maximized in the portal venous phase, the radiomic features were only extracted from CT images of the portal phase. We will use other phases to evaluate MMR protein status in the future. Thirdly, although the study cohorts were collected from two hospitals, multi-center cohorts are really needed to verify the generalization ability of the predictive model. Fourthly, at the same time, we should design prospective research to demonstrate the practicability of the radiomic model.

## Conclusion

We developed and validated a radiomic nomogram model that might be accurate to assess the MMR protein status of GC patients based on the radiomic signature and clinical features (age and CT-reported N stage). This prediction model is also a noninvasive detection model that can guide preoperative clinical management.

## Data availability statement

The datasets presented in this study can be found in online repositories. The names of the repository/repositories and accession number(s) can be found in the article/**Supplementary Material**.

## Ethics statement

This study was reviewed and approved by the Ethics Committee of the First Affiliated Hospital of Nanchang University. Written informed consent for participation was not required for this study in accordance with the national legislation and the institutional requirements.

## Author contributions

QZ, ZF, and LeL conceived the project and wrote the manuscript. YZ and FZ drew the ROI of CT images. XS, AW, and LiL participated in data analysis. YC and YT participated in discussion and language editing. JX and ZL reviewed the manuscript. All authors contributed to the article and approved the submitted version.

## Funding

This work was supported by the National Natural Science Foundation of China (No. 81860428), the Key R&D General Project of Jiangxi Science and Technology Department (20203 BBGL73187), and Youth Fund of Jiangxi Provincial Science and Technology Department (20202 BABL216051).

## Conflict of interest

The authors declare that the research was conducted in the absence of any commercial or financial relationships that could be construed as a potential conflict of interest.

## Publisher’s note

All claims expressed in this article are solely those of the authors and do not necessarily represent those of their affiliated organizations, or those of the publisher, the editors and the reviewers. Any product that may be evaluated in this article, or claim that may be made by its manufacturer, is not guaranteed or endorsed by the publisher.
