# Supplementary figures and images for: CT-based radiomic nomogram for preoperative prediction of DNA mismatch repair deficiency in gastric cancer
Source: Front Oncol. 2022 Sep 16;12:883109. doi: 10.3389/fonc.2022.883109 (PMC9523515; doi:10.3389/fonc.2022.883109)

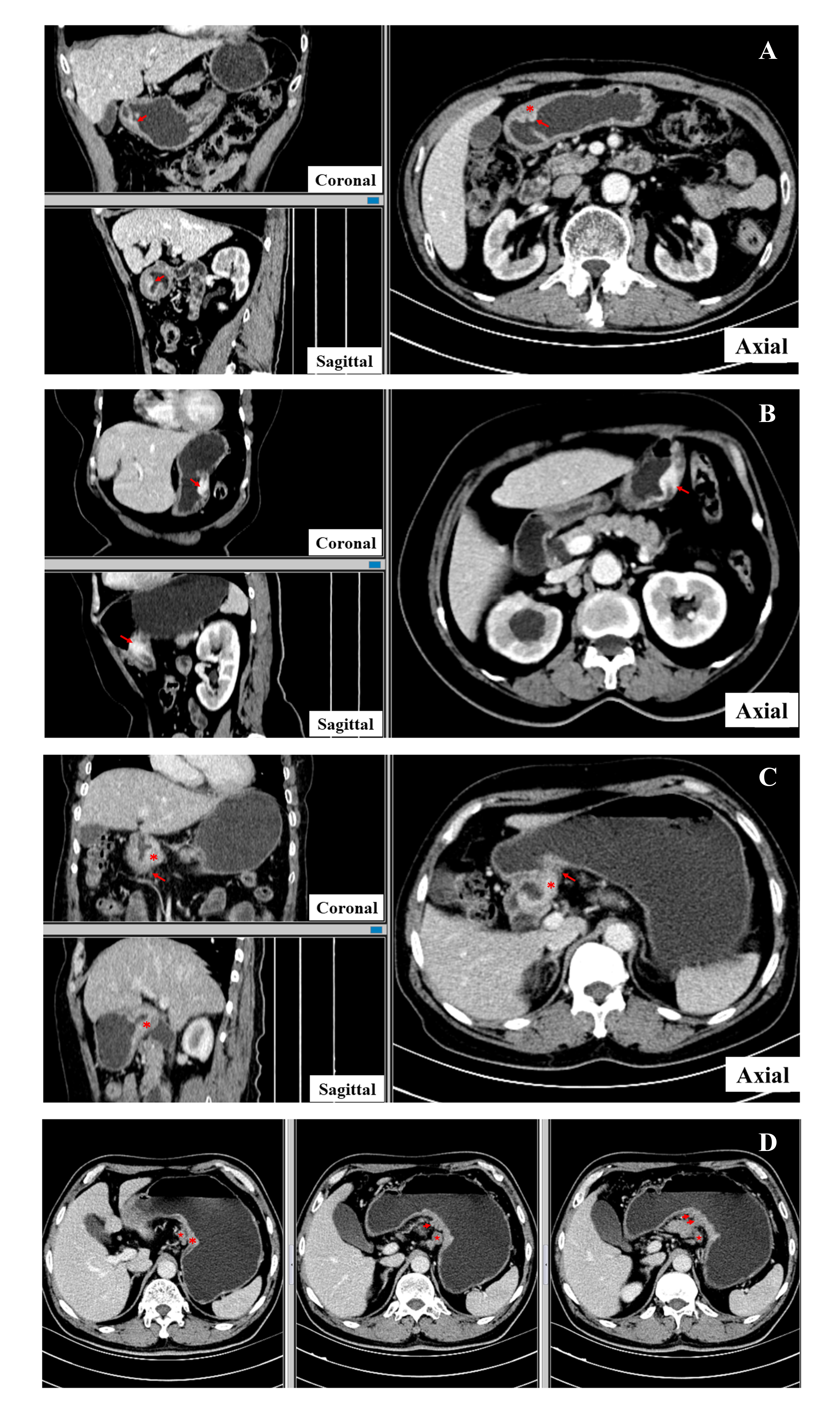

Supplement: Supplementary file 1 [file Image_1.tif]

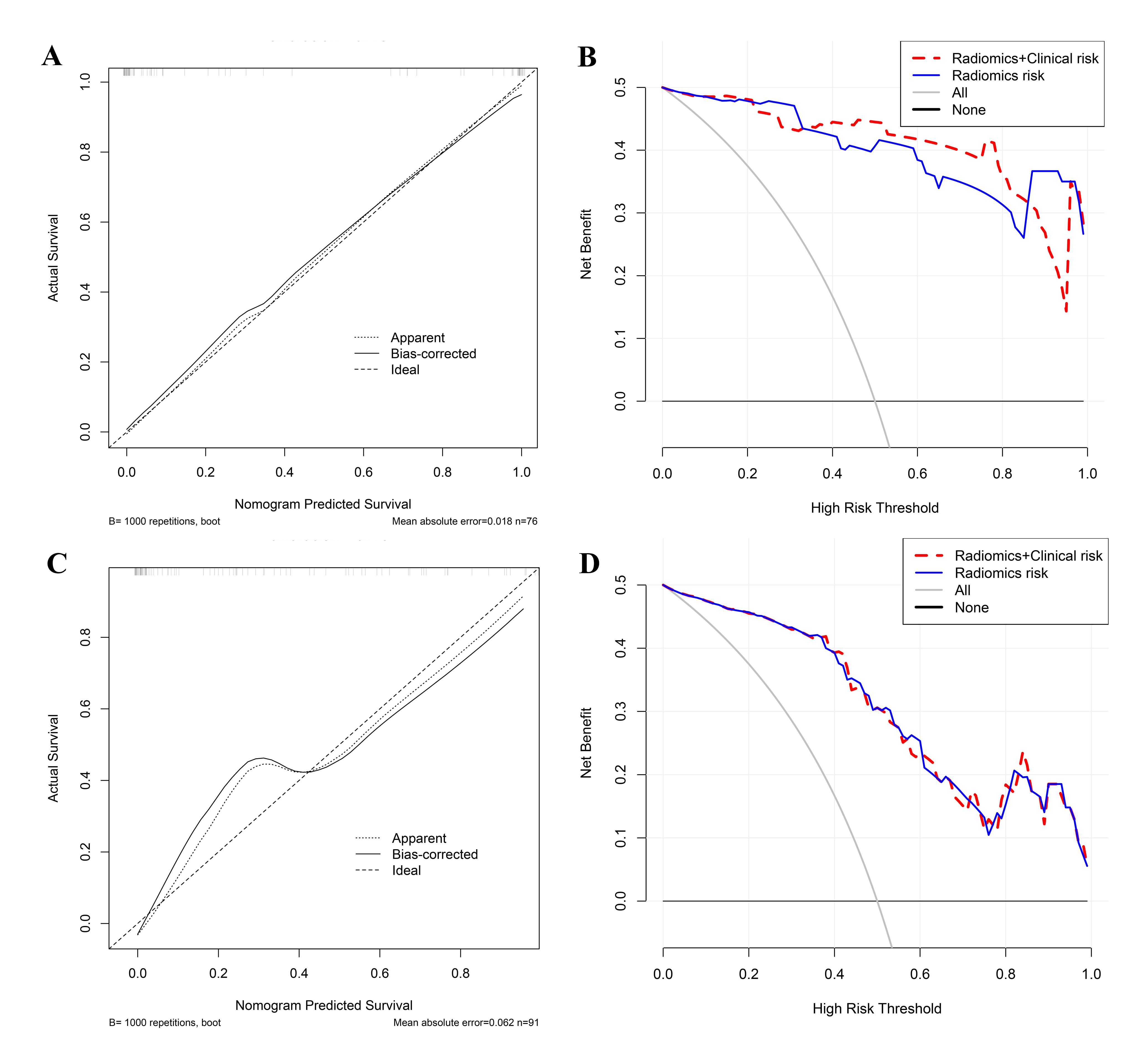

Supplement: Supplementary file 2 [file Image_2.tif]
